# Achieving equity in UHC interventions: who is left behind by neglected tropical disease programmes in Cameroon?

**DOI:** 10.1080/16549716.2021.1886457

**Published:** 2021-02-28

**Authors:** Makia Christine Masong, Kim Ozano, Marlene Siping Tagne, Marlene Ntchinda Tchoffo, Sharon Ngang, Rachael Thomson, Sally Theobald, Louis-Albert Tchuem Tchuenté, Estelle Kouokam

**Affiliations:** a Department of Social Sciences and Management, Catholic University of Central Africa, Yaoundé, Cameroon; b Department of International Public Health, Liverpool School of Tropical Medicine, Liverpool, UK; c Department of Sciences, Research Center for Schistosomiasis and Parasitology, University of Yaoundé 1, Yaoundé, Cameroon

**Keywords:** Equity, mass drug administration (MDA), missed populations, neglected tropical diseases (NTDs), marginalised communities

## Abstract

**Background**: The UN’s Sustainable Development Goals (SDGs) which pledge to leave no one behind for Universal health coverage (UHC) raise the importance of ensuring equitable health outcomes and healthcare delivery. As Neglected Tropical Diseases (NTDs) affect the most disadvantaged and hard to reach populations, they are considered a litmus test for Universal health coverage.

**Objective**: Here, we assess the challenges of implementing Mass Drug Administrations (MDAs) for schistosomiasis prevention and control, in a context of expanded treatment where both community and school-based distribution were carried out, assessing which groups are missed and developing strategies to enhance equity.

**Methods**: This is a qualitative study applying ethnographic observations, in-depth interviews (109) and focus group discussions (6) with key informants and other community members. Participants included community drug distributors, teachers, health workers, and implementing partners across four schistosomiasis endemic regions in Cameroon. Data collected were analysed thematically.

**Results**: Programme implementation gaps have created circumstances where indigenous farmers (originally from the region) and migrating farmers (not originally from the region known as ‘strangers’ and ‘farm hands’), women of reproductive age and school-aged children are continuously missed in MDA efforts in Cameroon. Key implementation challenges that limit access to MDA within this context include inadequate sensitization campaigns that don’t sufficiently build trust with different groups; limits in CDD training around pregnancy and reproductive health; lack of alignment between distribution and community availability and the exclusion of existing formal and informal governance structures that have established trusting community relationships.

**Conclusion**: Through identifying key populations missed in MDAs within specific contexts, we highlight how social inclusion and equity could be increased within the Cameroonian context. A main recommendation is to strengthen trust at the community level and work with established partnerships and local governance structures that can support sustainable solutions for more equitable MDA campaigns.

## Background

Neglected tropical diseases (NTDs) are responsible for approximately 150 000 deaths a year [1]. They cause life-altering morbidity, long-term disability, limit economic productivity [2,3]and lead to stigma, discrimination and social exclusion, especially for the most poor and marginalized [4–6]. The control and elimination of NTDs are directly linked to achieving several Sustainable Development Goals (SDGs) and the pledge to ‘leave no one behind’. Health Goal 3 includes a target to ‘end the epidemic’ of NTDs by 2030 (SDG Target 3.3). As NTDs affect the most disadvantaged and hard to reach populations, often without access to quality health services, they are considered a litmus test for Universal Health Coverage (UHC) and an equity ‘tracer’ [7–9]. Mass drug administration (MDA) of preventive chemotherapy (PC) for NTDs is a pathfinder for accelerated and cost−effective delivery of primary care [10]. Universal coverage to protect against and treat NTDs is not only about extending the coverage of ‘vertical’ disease programs but also about achieving a more equitable approach that mitigates against repeatedly leaving the same populations out of interventions [10,11]. Thus, there is a need to understand and bridge existing equity gaps through implementation research that explores program policies and implementation strategies and how these can be optimised to reach everyone.

### Mass drug administration for schistosomiasis in Cameroon

MDA is aimed at optimizing the largescale use of safe, single-dose drugs to reduce the extensive morbidity associated with 5 NTDs [12]. In Cameroon, MDA involves distributing drugs, donated by pharmaceutical companies, through school-based treatment of children [13–15]. However, evidence has shown that the current strategy which prioritises the treatment of school-aged children is no longer suitable for achieving schistosomiasis elimination goals [14]. Recently this has meant expanding access to MDA in highly endemic communities for at-risk adults as well as children through door-to-door community-based treatment programs [14]. The government in partnership with international implementing partners, non-government organisations, the education sector, community-based leaders and drug distributers is undertaking regular and systematic deworming twice a year alongside community-based distribution in endemic areas [15]. This research is collected within endemic regions that have been implementing both community and school-based deworming.

The 2016 statistics from Cameroon [12] showed that 5.7 million eligible people are still missed in MDA for schistosomiasis, Onchocerciasis and Lymphatic filariasis. More than 2 million children are in need of MDA treatment for schistosomiasis, known as bilharzia, however only 77% of those receive treatment [12]. In some health areas of Cameroon, such as Barombi Kotto the prevalence amongst school-aged children is over 25% [16]. A study investigating schistosomiasis prevalence amongst pregnant women in Cameroon found a prevalence of 31.9% [17]. School-aged children (5 to 14 years old), fishermen and farmers are known high-risk groups for Schistosomiasis infection as well as women and girls as gender roles mean that many of their daily activities include fresh water contact in the washing of clothes and dishes, fetching water for bathing, drinking and other activities [18]. However, disaggregated data on schistosomiasis prevalence of out of school children, pre-school children and at-risk adults including MDA coverage is lacking [19].

A core focus of the public health approach for UHC is preventive care and control. The NTD community, after many years of implementing community-directed approaches for MDA have key learnings that would benefit the UHC movement. Highlighting key populations that are continuously left behind due to programmatic, structural and contextual implementation bottlenecks and challenges will help improve equity within NTD programmes and achieve UHC [20]. Drawing on findings from this qualitative study, we assess key populations that are consistently missed during MDA campaigns for schistosomiasis in Cameroon and recommend strategies that could promote more inclusive and equitable coverage of treatment.

## Methods

### Study area and participants

**Figure 1.**
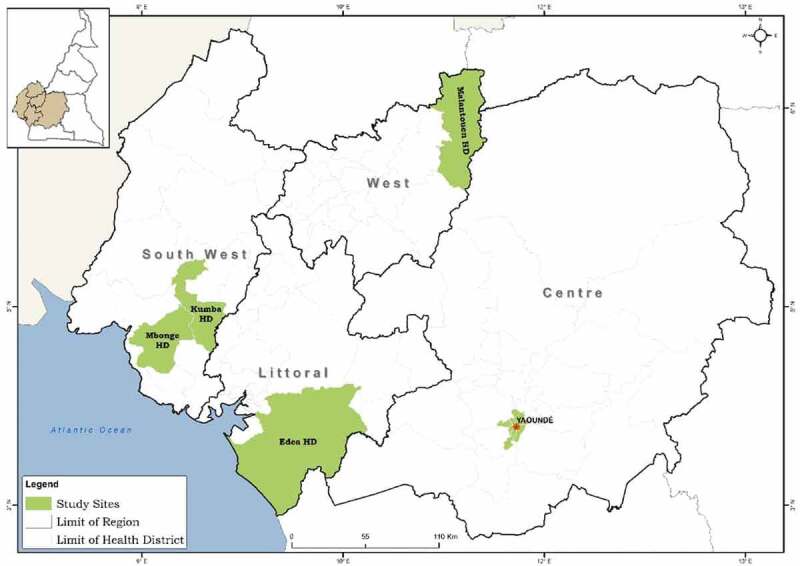
Excerpt from Cameroon map showing 5 health districts used for study

Our research was carried out in five health areas (Barombi Kotto, Barombi Mbo, Makenene, Yaoundé 1 and Edea 1) within five health districts (Mbonge, Kumba, Malanteoun, Edea, Yaoundé) in four regions of Cameroon (South West Region, West Region, Littoral Region, and Centre Region (Figure 1). Four of our five Health Districts were selected based on high endemicity of schistosomiasis and the intervention of parasitological activities as part of a larger multidisciplinary implementation cross-sectional research [21]. The fifth, Yaoundé, was selected as an active region for schistosomiasis with a high presence of implementing partners (at the strategic level) (Table 1). Participants were asked to reflect on previous MDA based in schools and any community-based distribution. Data were collected at different time points including during MDA and up to one month after the last MDA.

**Table 1.** Participant information showing different levels, characteristics and geographic location of different stakeholders

Level of Intervention	Stakeholders Met	Interviews			Gender		Total Number of stakeholders met	Sites
		Individual interview	FDG	Local level meeting	Male	Female		
Community	Community members (parents included)	57	3	0	40	58	98	Edea, Barombi Mbo, Barombi Kotto, Malanteoun
	Teachers	18	2	1	18	30	48	
	Community drug distributors	7	2	0	20	7	27	
	Health workers	12	0	0	5	7	12	
	Council workers/officials	4	1	0	10	4	14	
Intermediary	Health officials	5	0	0	3	2	5	Edea, Barombi Mbo, Barombi Kotto, Malanteoun
	Council workers/officials	6	0	0	4	2	6	
Strategic	Implementing partners	9	0	0	8	1	9	Yaoundé

### Ethnographic qualitative research

The ethnographic process [22] included 109 in-depth interviews, six focus group discussions (FDG), 1 local level meeting, and participant observation with resulting field diaries, photographs and informal discussions to better understand the realities of the different contexts (Table 1) [23]. The continuous presence of researchers in the field sites for a minimum of four weeks in each site facilitated rich ethnographic descriptions of social activities, analysis of community-level relationships [22], and their (potential) role in addressing equity gaps in MDA campaigns.

Participants included community members (adults eligible for MDA), parents of school-aged children eligible for MDA, teachers responsible for distributing drugs in schools, community drug distributors responsible for distributing drugs in communities, religious and village leaders (engaged or not in sensitisation efforts), health officials engaged in campaign sensitisation and coordinating distribution, council workers who were not health based but have potential to influence MDA, and strategic level partners such as NGOs and international implementing partners) (see Table 1).

Following an initial stakeholder analysis, key informants including council workers, health officials and implementing partners known to be involved in MDA were identified. These were approached through email or within known networks. Teachers were identified during field visits to schools who were then able to sign-post parents of children involved in the last MDA. Health workers based in community health facilities supported the engagement of CDDs involved in previous MDA campaigns. Community entry and engagement with community leaders’ led to the identification of community members and subsequent snowball sampling and sign-posting of other parents and community members further identified participants [24]. Purposive sampling was used to select the respondents based on a sample frame guided by the principle of maximum variation (including age, gender and location) to ensure a wide range of perspectives were included [25].

#### Analysis

**Figure 2.**
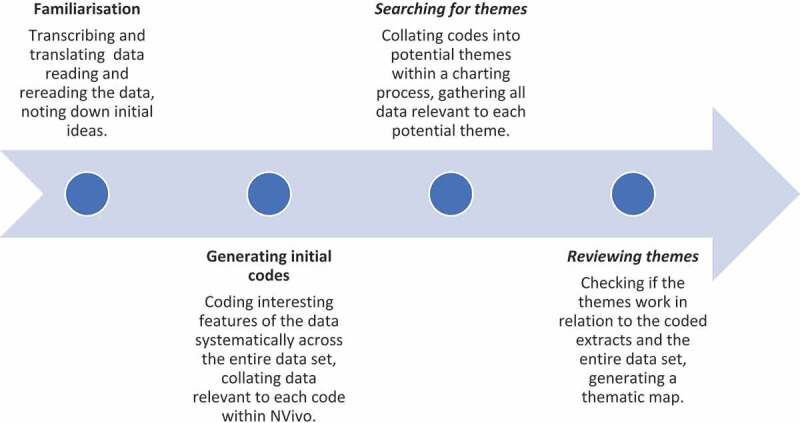
Summary of coding process adapted from Vaismoradi et al 2013 [28]

Interview length ranged from 30 minutes to 1 hour 30 minutes, and were conducted in either English, French or Pidgin-English or a combination. This depended on their location in either a French-speaking region (Littoral and Centre) or English speaking Region (Barombi Kotto and Barombi Mbo). Interviews were translated to English, if necessary, after transcription in the local language [26]. As the researchers who conducted the interviews spoke the local language, French and English there were minimal issues in the translation of data. The three social scientists involved in data collection quality checked translations by listening to audio recordings and sampling sections of the transcriptions for accuracy. The data were organized by themes using a thematic framework approach summarised in Figure 2 [2–26]. A coding framework was developed collaboratively within the research team and applied to the data independently by members of the research team using Nvivo 11 software. Once all data had been coded, similarities and differences within each code were reviewed to develop thematic charts with consideration of characteristics, such as age, sex, level of education, work experience and geographical location. Once the charts had been completed, the data were discussed in detail by the research team to interpret descriptive and explanatory accounts of each emergent theme [27].

#### Ethical approval and considerations

Ethical approval for our research was granted by the Cameroonian National Ethics Committee for Research on Human Health (approval no. 2016/11/838/CE/CNERSH/SP), the Division of Health Operations Research within the Cameroonian Ministry of Public Health (approval no. 631-03.17) and from the Liverpool School of Tropical Medicine (15.043). The objectives of the study were explained to participants through information notice sheets read out and translated to English, French or pidgin-English, and in some cases the local dialect. Informed written consent or a thumb print was obtained from all participants.

## Results

**Figure 3.**
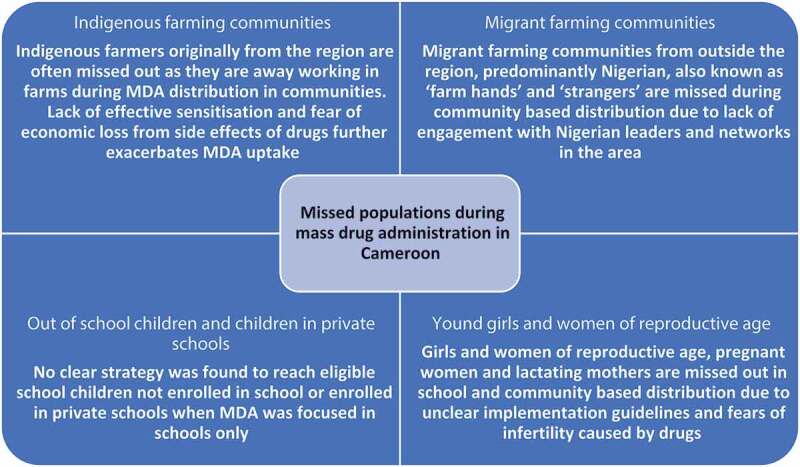
Missed populations during mass drug administration in Cameroon

The key populations that emerged from the analysis as being repeatedly missed in MDA campaigns are indigenous farmers (originally from the region) and migrant farming communities (farmers from outside the region also known as ‘farm hands’ and ‘strangers’), women of reproductive age and school aged children. The reasons for being missed and potential existing mechanisms to close this gap in coverage are discussed in sub-themes and summarized in Figure 3 and Table 2.

**Table 2.** Results table to show clear problem-solution linkages and commonality across results

Who is missed	Why are they missed	Who can help	How can they help
Farming communities	Absence during distribution Insufficient sensitization (time and content) Distribution period	National Program implementation (National Programs for the specific NTDs)	By adapting programs to community calendars. By upgrading sensitization efforts through increased time and more informative sensitization (side effects, reasons for medicines, why needed, effects of not taking, methods of contamination and transmission)
Migrant populations	Absence of migrant communities during distribution Context/cultural differences leaving populations left out in sensitization and distribution	National Program implementation Quarter heads/village leaders identified in different communities	Programs should engage and train community leaders so they may identify people currently left out and conduct sensitization and distribution to facilitate understanding and acceptance of why medicines are needed. Ensure better understanding migrant patterns of workers
Young girls and women of reproductive age	Pregnant and lactating mothers not offered Fear and mistrust due to insufficient sensitization Training of CDDs in relation to this group of women Include or revisit national Policy on treating women and young girls	National Policy/Program implementation	Policy specifications on the distribution of drugs to pregnant women by drug distributors should be updated and implemented consistently according to the World Health Organization recommendation Information on administering medicines to pregnant women shared with health districts and included in the training on CDDs (distribution of praziquantel for Schistosomiasis control specifically). Content of sensitization messages should include information on safety, including the need for medicines and addressing side effects, to dispel fears around impacts on pregnancy and fertility of young girls.
Children	Out school of children not met Private (and least populated) schools mostly left out	National Program policy National Program planning. Education Sector partners. Health Sector partners	Include clear plan for distribution of medicines to out of school children during school-based distribution, or if already existing, practiced and monitored for effectiveness. Program planning and implementation to identify mechanisms to reach private schools being left out

### Farming communities

Barombi Kotto in the South West of Cameroon has a high number of migrant farmers that migrate from Nigeria and other areas of Cameroon for work. Barombi Kotto consists of a mainland area and island with a total population of about 2185 people [29]. From the data, it became apparent that indigenous and migrant farming communities are largely left out of MDA campaigns due to; the mobility of their lives and work, a lack of engagement with key community actors and socio-cultural differences from other Cameroonians. Contextual factors were reported to impact on this migrant community’s ability to access and accept medicines for NTDs.

### ‘Strangers’ and ‘farm hands’

The majority of the inhabitants on the mainland (the most populated with over 1500 people) are termed ‘strangers’ by local people living in Kotto because they are not originally from the Kotto village. These ‘strangers’ consist of; Cameroonians from other regions; Nigerians who are settlers attracted by the generous cocoa farming and harvests and; migrant ‘Farm hands’ who are unpaid farm workers but receive food and clothes with paid trips back to Nigeria during cultural festive periods in exchange. During peak harvest periods the farm helpers can be away for up to two weeks at a time, where they inhabit farmhouses and rarely come down to the village. Most of their decisions are influenced by their ‘sponsors’, a term used for the owners of the farms on which they work, such as feeding, health and sanitation, and even periods of visit to their original hometowns. A health official describes the mobility of the Nigerian population;

… people come … here is a zone with many foreigners, especially Nigerians who migrate home once or twice a year … during December most of them go to Nigeria and come back in April or May … others leave and go in April and only come back at the end of May or in June to harvest their products … Male Health Official, IDI Kotto

During the cocoa farming season, it was reported that over 800 Nigerians come to this village [29], with each farmer having up to 10 unpaid ‘Farm Helpers’ all from Nigeria. In July and December, the Nigerian population usually returns to Nigeria. A community leader that represents Nigerians explained this is often why they are missed out in MDA;

… during the period when this deworming took place, we were in Nigeria, we travelled to Nigeria … Nigerian Male Community Leader, IDI Barombi Kotto

The migrant farmers from Nigeria indicated that they had not received treatment for schistosomiasis whilst in Nigeria and stated that in most of the States they came from, MDA for schistosomiasis was not performed routinely. MDA took place firstly in July, the rainy season and the best time for planting, when most of the farmers spend long periods in the bushes (with the farm hands spending longer since it is their job to stay back and complete the work). The second deworming MDA was carried out between October and December when farmers and other migrants in the community had moved to their original communities and countries for holidays to return during harvesting season, and so were possibly missed out again.

### Governance structures that support local populations

Barombi Kotto has strong local governance structures, where each quarter has a quarter head and leaders for men, women and youths. In addition, for the Nigerian community, there is an appointed Nigerian Leader who is the president of the Nigerian Union and represents the interests of the Nigerian population in the village. The president is elected by the resident Nigerian population and issues concerning discipline, sanitation and health are coordinated by him with the assistance of the quarter heads, and he reports to the Kotto Chief. Nigerian ‘strangers’ and ‘farm helpers’ are mostly concentrated in the Bule Quarter. The involvement of these governance actors reflects on the participation of Nigerians in health interventions.

These local leaders were proposed as the most effective persons to do follow up of those who do not want to participate and convince the workers within their area to participate or explain their concerns. This was discussed in a FGD with community women;

… especially when the chief comes to tell them … women get vaccinated … Sometimes we even repel vaccinators who go door to door … sometimes it is a leader who manages the conflicts between distributors and parents, to make them let their child drink the medicine … Female Community Member, FGD Edea

All these leaders are respected and their decisions influence that of the community members. They are also able to easily identify areas not covered during MDA and in some instances identify persons who are missed.

During a schistosomiasis testing campaign we participated in, the sensitization did not reach most people and they refused to participate, but when we approached these leaders explaining our objective of the campaign, within a day over 150 people especially from the Nigerian community willingly participated in the tests, unlike the barely 45 people we had been able to test for schistosomiasis in the first few days. Ethnographic Field Diaries, Barombi Kotto

These leaders are strategic links between the health system and the community; they are well aware of the movement or mobility plan of the community members and have the potential to advise on when to conduct deworming interventions. This demonstrates how differing contexts need to be considered and interventions tailored to suit communities or large population groups such as migrating farmers who are often missed.

### Barriers to participation

#### Insufficient sensitization processes

A common challenge in all research sites was delivering effective sensitization, where CDDs and community members mentioned this was limited;

we did not have much time to raise awareness … we had little time, few days to sensitize. We didn’t have nearly two days, and it was very small Male CDD, Edea

… the time they spent here was very small … We must be informed before the distribution time so that we organize ourselves accordingly and knowing that we are expected to take drugs. FGD with Men Community members, Makenene

In addition to the length of time for sensitizing community members, sensitization must also be done in a way that effectively engages communities and improves their understanding otherwise they may listen to education campaigns but not take the medicines if the information is not understood;

… Some of them listen when the information reaches them, but I don’t know, some even pretend to collect it [medicine], then they go and keep it and don’t drink it … FGD with Male Community Leaders, Barombi Kotto

#### Fear of economic loss from taking of medicines distributed during MDA campaigns

Findings revealed some farmers felt that the drugs given them, specifically Praziquantel and Mebendazole for Schistosomiasis and STH, slowed them down and reduced functioning in their farms especially when given in the morning.

… besides, some people drank the medicine and found themselves in the hospital or weak at home. You see it confirms people’s fear … it will stop your work and when it is the season like this you lose a lot. FGD Male Community Members, Makenene

… they [farmers] complained, taking these medicines before going to work on the farms slowed them down, at times missing a whole day of farm. People advised their friends after having taken the drug to keep away from it or to take on the day when they knew they didn’t want to work. Informal conversations, Field Journals, Barombi Kotto and Barombi Mbo

## Young girls and women of reproductive age

Girls and women of reproductive age, pregnant women and lactating mothers are missed out in MDA campaigns because of programme implementation gaps in the cases where policies exist for the treatment of this specific group but are not followed or where no specific national policies exist on this issue. Community Drug Distributors (CDDs) remained untrained in this area and reported uncertainty about giving medicine to pregnant women.

… I didn’t know which the right way was, they didn’t tell us … I had mentioned that issue about the women with small babies and pregnant women, those were some little things I did not know about and I had some difficulties in the field …” Female CDD, IDI Barombi Kotto

### Fear and mistrust of medicines as a cause for infertility and forced sterility

From our ethnographic studies, we found this gap of knowledge, and doubts among distributors, have resulted in this group of women refusing treatment when offered because of fear of harming their unborn babies or new-borns.

… several lactating mothers and pregnant women refused taking the drug when it was offered and in an instance a woman told of the story of another pregnant woman who had lost her baby after drinking Praziquantel. In another instance, a distributor refused giving a pregnant woman with haematuria, saying he wasn’t sure and feared taking blame adverse effects Ethnographic Field Diaries, Edea

Implementation gaps around limited sensitization messages also encourage the existence of myths or mistrust of why the medicines are given. Local beliefs around fertility were encountered in a mixed focus group (both men and women) on perceptions of deworming campaigns;

… it seems the woman who does not give birth is not given … seems they give it only to women of childbearing age and fertile. I never understood why. Or because we no longer want children in Cameroon, or because we want to destroy our bellies, I do not know Mixed FGD, Females, Parents, Edea

## Children left out during MDA school campaigns

No clear strategy was found to reach eligible children not enrolled in schools or enrolled in private schools (mostly schools not registered with the Ministry of Basic Education) when the focus of MDA was purely within schools. In many cases, teachers did not manage to reach these children.

… no they don’t take. The children of that age in the quarters not attending school, don’t come to ask for theirs … we don’t look for them Female Head Teacher, KII Edea

all schools are not involved … just the most populated schools … other children within the age group selected who were not enrolled … there was not a clear um, clear strategy to get the non-enrolled children, so we just announce it for those who are not enrolled in school to come to schools and collect the medication but mostly they were will not come. Female Health Official, KII Regional Delegation of Public Health

Table 2 outlines the groups who are missed, the reasons behind this, and the potential people and strategies that could promote equity and inclusion in MDA campaigns.

## Discussion

Our findings show that program implementation gaps have created circumstances where some people/groups are repeatedly missed in MDA efforts in Cameroon. The reasons we have identified within the Cameroonian context include poor or inadequate sensitization campaigns, limits in CDD training and capacity, lack of alignment between distribution and availability of many community groups and inequitable distribution from applying a one size fits all approach that is not tailored to the realities and challenges of specific groups. We have shown that migrant farming groups, indigenous farmers, women and girls and children not in school are being consistently missed out. Our findings confirm the importance of understanding the challenges faced by marginalized groups and designing MDA campaigns to meet the needs of those left behind as found in other studies [30,31].

Building the evidence base on those left behind, through NTD programs can provide insights on marginalised populations. The objective of accessing all populations can be met through focusing on equity implementation challenges and learning across contexts [30]. The breakdown of ‘all populations’ into different specific groups shaped by gender, political, economic and other relevant context-specific axes of inequity cannot be over-emphasized. The realities of these different groups, many of whom are indeed not even considered as stakeholders in NTD interventions, encourage their exclusion during health interventions. Through identifying key populations and specific contexts, we throw light on where social inclusion and equity could be increased within the Cameroon context. One method clearly identified here is improved program collaboration with local governance structures which have established trust in the community in order to support more equitable and sustainable MDA campaigns. As NTDs are a litmus test for UHC, there is a clear need to focus on equity in health including precision and fairness of health intervention delivery which should be people-centred, inclusive, participatory and responsive to differing contexts and specificities [32].

### Considering gender and its impact on MDA participation

SDG 5 aims to address the existing situation of gender inequality where women and girls are deprived of basic rights and opportunities to health care. We highlight here the case of schistosomiasis where women in their reproductive years spend more than 25% of their time pregnant or lactating [13,33]. With the irregularities existing in the distribution of praziquantel to pregnant or lactating women, these women are constantly missed [13,34]. A clear treatment strategy must be developed for pregnant women and girls of reproductive age [35] as their participation in MDA, though prescribed by the WHO, remains an issue in most implementing programs [33,36]. For the case of Cameroon, the Schistosomiasis national program [37] is waiting for additional research and confirmation of treatment safety for pregnant women before a clear national policy is set, highlighting the urgency of additional research and/or alignment with global evidence to support national program decision-making in endemic countries.

Our results demonstrate that girls and women lack trust in medicines and have fears of the impact they may have on their reproductive capacity; or if pregnant, worries that they may miscarry [38]. This fear is not only Cameroon specific but also reported elsewhere including Tanzania and Haiti [3,39]. NTD programme planners need to provide adequate time for sensitization and space to collaboratively design educational messages that resonate with women and their fears around fertility and harm to unborn children. Collaborative multi-sectoral working, including with health professionals working in sexual and reproductive and maternal and child health will be strategic to provide advice and education to girls and women and to facilitate trust in medicines and clearly explain when it is safe to participate in MDA [40].

### Multi-sectoral action to promote more equitable MDA distribution

During school deworming campaigns, our study found that some children are missed due to the lack of deworming coverage in some private schools and children who do not attend school [41]. In addition, refusals due to poor sensitization are an issue [41,42]. There is a need for NTD programs to form multi-sectoral partnerships with the education sector and private sector schools to address this and to collaboratively sensitize and motivate parents and children in school, and out of school, to engage with health interventions. Currently, this responsibility is mostly left to teachers, who have highlighted such sensitizations are better placed, and will be more respected, if carried out by health personnel. NTD programs and funders need to support teachers and others in the roles expected of them and ensure joint planning as the timing for MDA is limited and teachers don’t get a lot of time in advance to sensitize children and families before MDA is conducted [41]. The engagement of decentralized councils and other local structures within the community will also support the involvement of other multi-sectoral stakeholders.

### Embracing formal and informal social networks

Labour migrants are common in Cameroon and elsewhere in Sub-Saharan Africa and are often missed in health interventions which are not responsive to their social realities and situations [43]. There is a need to understand the realities of different populations [31] and ensure that policy and practice (and hence training) encourages front line providers to develop responsive context/group-specific strategies.

Our findings highlight opportunities for change and strategic partnership at the local level for better equity and sustainable program implementation. Whilst it is recognised that there is some level of engagement with local networks, for each of the missed groups identified here, formal and informal trusted community structures exist but remained neglected or invisible in the implementation process. Their lack of involvement is a missed opportunity to decrease inequities in NTD control and elimination and other health programmes. NTD programme implementers could adjust institutional behaviours by developing stronger links with local governance and social support networks to build on existing social capital [44]. Partnering with local-level stakeholders could strengthen sensitization messages to address concerns and myths and contribute to monitoring of health-related activities and behaviours within communities. Integrated training that includes people from governance structures, community health workers, local authorities like quarter heads, councils, and local health centres who have local knowledge, insights and strengths is likely to improve partnerships and solution development and promote sensitization processes that embrace contextualized interventions as found in here and in other African contexts [31].

### Limitations and future research areas

One weakness is that we did not collect data with NTD implementers, however their perspectives are often captured in evidence-based policies [45] leaving gaps around understanding experiences from marginalized groups. We captured and shared local voices of the often ‘invisible’ populations by employing multiple methods including ethnographic research which allowed us to triangulate and capture the social structures and bottlenecks that exist at community level and have clear implications for equity within health policies. With the evidence presented here, NTD actors will be able to take steps to understand the logistical, financial and programmatic changes required to reach these at-risk populations in order to meet schistosomiasis elimination goals. Furthermore, there is a lack of statistical data on prevalence and coverage for each identified group, collecting disaggregated data for missed population groups identified by qualitative research is a recommendation for future research related to achieving UHC goals.

## Conclusion

Policy gaps and implementation challenges are creating inequities in the delivery of MDA for neglected tropical diseases which affect the poorest and most marginalised communities in Cameroon. The large numbers of ‘invisible’ farmers and migrant populations that are at risk of disease are unnecessarily missed out of health interventions due to a lack of engagement with trusted governance structures. The treatment and prevention of schistosomiasis for women of reproductive age and children are being disregarded by policymakers because of a lack of clarity around evidence produced at the global level and the resulting distrust that comes from uncertainty. Here we suggest small steps regarding programme implementation, policy review and engagement of local level multi-sectoral stakeholders who have solutions and thus can promote equity and inclusion within health programs in order to achieve Universal Health Coverage.

## Paper context

Much research has taken place to understand neglected tropical disease programmes and associated implementation challenges in Cameroon, but there exists limited research that examines who is consistently left behind by these annual health interventions. In five schistosomiasis endemic communities composed of rich and diverse cultural and socio-economic backgrounds where migrant farming families, women of reproductive age and school-aged children make up a huge part of the population, we explore why they are constantly missed.

## References

[1] Liese B, Rosenberg M, Schratz A. Programmes, partnerships, and governance for elimination and control of neglected tropical diseases. Lancet. 2010;375:67–11. 20109865 10.1016/S0140-6736(09)61749-9

[2] Manderson L, Aagaard-Hansen J, Allotey P, Gyapong M and Sommerfield J. Social research on neglected diseases of poverty: continuing and emerging themes. PLoS Negl Trop Dis. 2009;3:e332. 19238216 10.1371/journal.pntd.0000332 PMC2643480

[3] Theobald S, MacPherson EE, Dean L, et al. 20 years of gender mainstreaming in health: lessons and reflections for the neglected tropical diseases community. BMJ Glob Health. 2017;2:e000512. 10.1136/bmjgh-2017-000512 PMC5687534 29177100

[4] Weiss MG, Utzinger J. Stigma and the social burden of neglected tropical diseases. PLoS Negl Trop Dis. 2008;2:e237. 18478049 10.1371/journal.pntd.0000237 PMC2359851

[5] Bailey F, Eaton J, Jidda M, et al. Neglected tropical diseases and mental health: progress, partnerships, and integration. Trends Parasitol. 2019;35:23–31. 30578149 10.1016/j.pt.2018.11.001

[6] Bangert M, Molyneux DH, Lindsay SW, et al. The cross-cutting contribution of the end of neglected tropical diseases to the sustainable development goals. Infect Dis Poverty. 2017;6:73. 28372566 10.1186/s40249-017-0288-0 PMC5379574

[7] Molyneux DH, Savioli L, Engels D. Neglected tropical diseases: progress towards addressing the chronic pandemic. Lancet. 2017;389:312–325. 27639954 10.1016/S0140-6736(16)30171-4

[8] Smith J, Taylor EM, Addiss DG. What is next for NTDs in the era of the sustainable development goals? PLoS Negl Trop Dis. 2016;10:e0004719. 27387209 10.1371/journal.pntd.0004719 PMC4936684

[9] Fitzpatrick C, Engels D. Leaving no one behind: a neglected tropical disease indicator and tracers for the sustainable development goals: box 1. Int Health. 2016;8:i15–8. 26940304 10.1093/inthealth/ihw002 PMC4777229

[10] Engels D, Daumerie D. Investing to overcome the global impact of neglected tropical diseases: third WHO report on neglected tropical diseases. In: WHO, editor. Geneva: World Health Organization’s Department of Control of Neglected Tropical Diseases; 2013.

[11] Hotez PJ, Kamath A, Cappello M. Investing to overcome the global impact of neglected tropicaldiseases: Third WHO report on neglected diseases 2015, p. 11-36.

[12] Uniting to Combat Neglected Tropical Diseases. Taux de couverture des traitements de masse pour les MTN. Le Cameroun et les maladies tropicales négligées. London: Uniting to Combat NTDs; 2016.

[13] Rilkoff H, Tukahebwa EM, Fleming FM, et al. Exploring gender dimensions of treatment programmes for neglected tropical diseases in Uganda. PLoS Negl Trop Dis. 2013;7:e2312. 23875047 10.1371/journal.pntd.0002312 PMC3708858

[14] Tchuem Tchuenté LA, Ombede DRE, Noumedem CD, et al. Prospects for the elimination of schistosomiasis and soil-transmitted helminthiasis: exploring disease trends through time at the Barombi crater lakes, South-West Cameroon. Parasitology. 2018;145:1700–1714. Epub 2018/09/24. 30246664 10.1017/S0031182018001531

[15] Tchuem Tchuenté L-A, Stothard JR, Rollinson D, et al. Precision mapping: an innovative tool and way forward to shrink the map, better target interventions, and accelerate toward the elimination of schistosomiasis. PLoS Negl Trop Dis. 2018;12:e0006563. 30071014 10.1371/journal.pntd.0006563 PMC6071947

[16] Nono JK, Kamdem SD, Netongo PM, et al. Schistosomiasis burden and its association with lower measles vaccine responses in school children from rural Cameroon. Front Immunol. 2018;9:229. PubMed PMID: 30356757. 30356757 10.3389/fimmu.2018.02295 PMC6189399

[17] Tonga C, Bayoi CN, Tchanga FC, et al. Schistosomiasis among pregnant women in Njombe-Penja health district, Cameroon. J Infect Developing Countries. 2019;13:1150–1158. 10.3855/jidc.11767 32088703

[18] Ozano K, Dean L, MacPherson EE, et al. Discussion paper: the gender dimensions of neglected tropical diseases: access and delivery partnership; 2019 [cited 2019 Dec 6th]. Available from: https://adphealth.org/upload/resource/2523_ADP_Discussion_Paper_NTDs_211119_web.pdf

[19] Faust CL, Osakunor DNM, Downs JA, et al. Schistosomiasis control: leave no age group behind. Trends Parasitol. 2020;36:582–591. 32430274 10.1016/j.pt.2020.04.012 PMC7905337

[20] World Health Organization. Towards universal coverage for preventive chemotherapy for neglected tropical diseases: guidance for assessing “who is being left behind and why”. Geneva: WHO; 2017.

[21] Countdown. Calling time on neglected tropical diseases; 2020 [cited 2020 Aug 16]. Available from: https://countdown.lstmed.ac.uk/

[22] Paillet A. The ethnography of ‘particularly sensitive’ activities: how ‘social expectations of ethnography’ may reduce sociological and anthropological scope. SAGE J. 2013;14:126–142.

[23] Lambert H. Anthropology in health research: from qualitative methods to multidisciplinarity. BMJ. 2002;325:210–213. 12142313 10.1136/bmj.325.7357.210 PMC1123726

[24] Williamson K, McGregor J. The sage handbook of qualitative research. In: Norman K, Denzin D, editors. Library & information science research 28. Thousand Oaks, CA: Sage; 2005, p. 797, 844-845.

[25] Ritchie J, Lewis J, Nicholls CM, et al., editors. Qualitative research practice: a guide for social science students and researchers. London: SAGE Publications Ltd; 2013.

[26] International Rescue Committee. Qualitative data transcription and translation. New York, USA: IRC; 2018.

[27] Patton M. Qualitative research and evaluation methods. California : Sage; 2001.

[28] Vaismoradi M, Turunen H, Bondas T. Content analysis and thematic analysis: implications for conducting a qualitative descriptive study. Nurs Health Sci. 2013;15:398–405. 23480423 10.1111/nhs.12048

[29] Mbonge Health District. Malaria census report. In: Mbonge Health District, editor. Yaoundé: Ministry of Public Health; 2016, p. 41.

[30] Krentel A, Gyapong M, Ogundahunsi O, et al. Ensuring no one is left behind: urgent action required to address implementation challenges for NTD control and elimination. PLoS Negl Trop Dis. 2018;12:e0006426. 29879105 10.1371/journal.pntd.0006426 PMC5991654

[31] Dean L, Page S, Hawkins K, et al. Tailoring mass drug administration to context: implementation research is critical in achieving equitable progress in the control and elimination of helminth neglected tropical diseases in sub-Saharan Africa. Int Health. 2016;8:233–234. 27481833 10.1093/inthealth/ihw031

[32] World Health Organization Regional Office for Western Pacific. People centered health care: a policy framework. Geneva: WHO; 2007.

[33] World Health Organization. Report of the WHO informal consultation on the use of praziquantel during pregnancy/lactation and albendazole/mebendazole in children under 24 months. Geneva: WHO, 2003.

[34] Ozano K, Dean L, Yoshimura M, et al. A call to action for universal health coverage: why we need to address gender inequities in the neglected tropical diseases community. PLoS Negl Trop Dis. 2020;14:e0007786. 32163416 10.1371/journal.pntd.0007786 PMC7067373

[35] Gyorkos TW, Montresor A, Belizario V, et al. The right to deworming: the case for girls and women of reproductive age. PLoS Negl Trop Dis. 2018;12:e0006740. 30462641 10.1371/journal.pntd.0006740 PMC6248892

[36] Hotez PJ. Empowering Women and. Improving female reproductive health through control of neglected tropical diseases. PLoS Negl Trop Dis. 2009;3:e559. 19936248 10.1371/journal.pntd.0000559 PMC2775907

[37] Program Nationale de la Lutte Contre la Schistosomiase et les Helminths du Cameroun. Plan strategique 2005–2010. Yaoundé: Ministére de La Santé Pulique, Cameroun; 2005.

[38] St-Denis K, Blouin B, Rahme E, et al. Ruling out early trimester pregnancy when implementing community-based deworming programs. PLoS Negl Trop Dis. 2020;14:e0007901. 31999690 10.1371/journal.pntd.0007901 PMC6991962

[39] Krentel A, Fischer PU, Weil GJ. A review of factors that influence individual compliance with mass drug administration for elimination of lymphatic filariasis. PLoS Negl Trop Dis. 2013;7:e2447. 24278486 10.1371/journal.pntd.0002447 PMC3836848

[40] Gyapong M, Theobald S. The sexual and reproductive health issue you’ve probably never heard of . … 2015; 2020 [cited 2020]. Available from: https://www.opendemocracy.net/5050/margaret-gyapong-sally-theobald/sexual-and-reproductive-health-issue-you%E2%80%99ve-probably-never-hear

[41] Musuva R, Matey E, Masaku J, et al. Lessons from implementing mass drug administration for soil transmitted helminths among pre-school aged children during school based deworming program at the Kenyan coast. BMC Public Health. 2017;17:575. 28615011 10.1186/s12889-017-4481-7 PMC5471907

[42] Dean L, Tolhurst R, Nallo G, et al. Neglected tropical disease as a ‘biographical disruption’: listening to the narratives of affected persons to develop integrated people centred care in Liberia. PLoS Negl Trop Dis. 2019;13:e0007710. 31490931 10.1371/journal.pntd.0007710 PMC6750611

[43] Hansen J, Chaignat CL. Neglected tropical diseases: equity and social determinants. In: WHO, editor. Equity, social determinants and public health programmes. Geneva, Switzerland: WHO; 2010, p. 135–157.

[44] Uniting to Combat Neglected Tropical Diseases. Reachin a billion, ending neglected tropical diseases: a gateway to universal health coverage; 2019 [cited 2019]. Available from: https://unitingtocombatntds.org/reports/5th-report/

[45] Head BW. Reconsidering evidence-based policy: key issues and challenges. Policy Soc. 2010;29:77–94.

